# Supplementary material for: Inference technique for the synaptic conductances in rhythmically active networks and application to respiratory central pattern generation circuits
Source: bioRxiv. 2024 Aug 13:2024.08.12.607656. Preprint. [Version 1] doi: 10.1101/2024.08.12.607656 (PMC11343156; doi:10.1101/2024.08.12.607656)
Supplement: Supplementary file 1 [file NIHPP2024.08.12.607656v1-supplement-1.pdf]

bioRxiv preprint doi: https://doi.org/10.1101/2024.08.12.607656; this version posted August 13, 2024. The copyright holder for this preprint (which was not certified by peer review) is the author/funder. This article is a US Government work. It is not subject to copyright under 17 USC 105 and is also made available for use under a CC0 license.

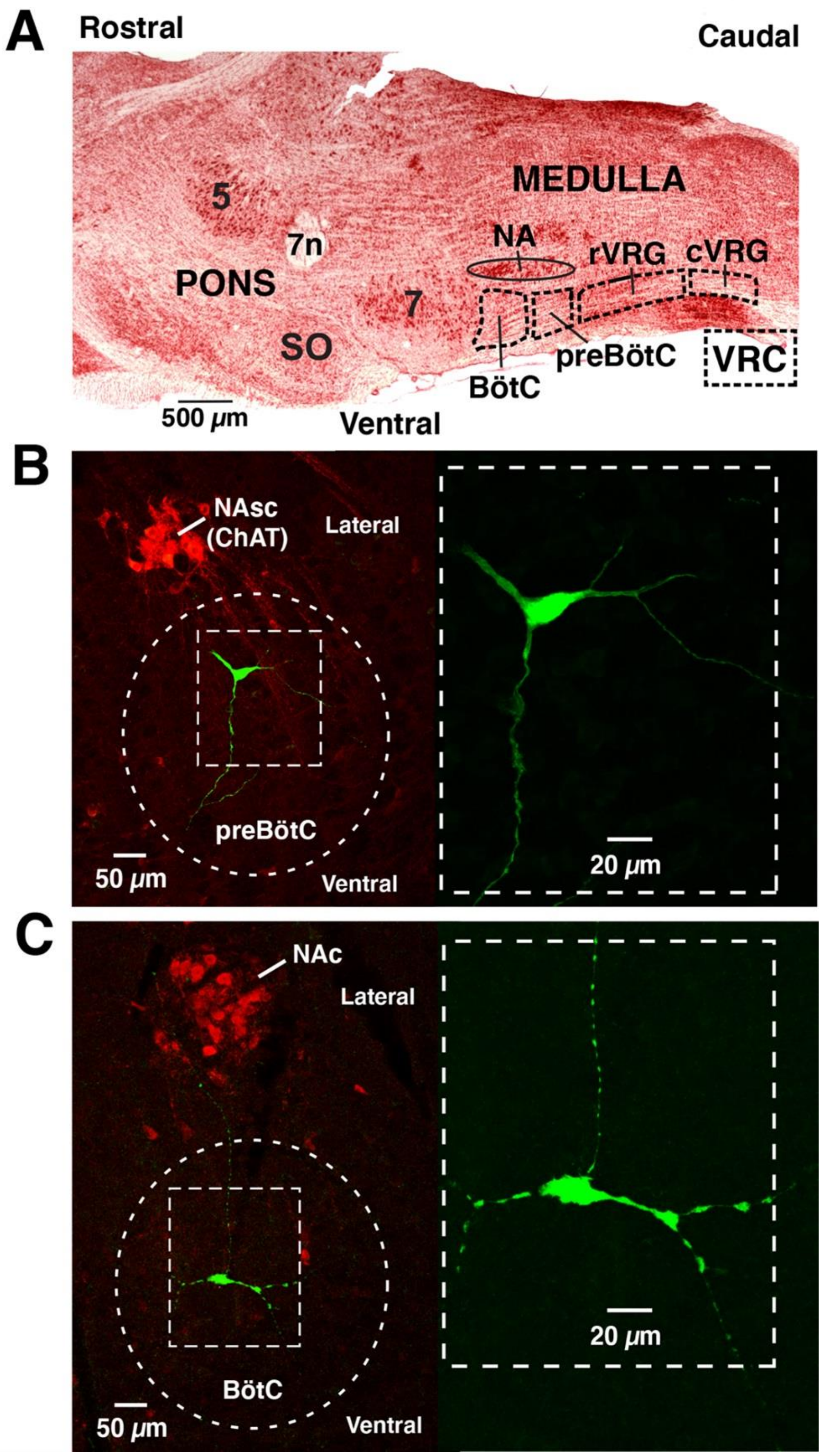

Supplemental Figure 1. **Respiratory regions of the brainstem ventrolateral medulla and intracellular recording sites. A**. Parasagittal view (neutral red stain) of the lateral medulla indicating regions of the ventral respiratory column (VRC) and locations of the preBötC and BötC regions of the VRC in the

bioRxiv preprint doi: https://doi.org/10.1101/2024.08.12.607656; this version posted August 13, 2024. The copyright holder for this preprint (which was not certified by peer review) is the author/funder. This article is a US Government work. It is not subject to copyright under 17 USC 105 and is also made available for use under a CC0 license.

ventrolateral medulla, ventral to nucleus ambiguus (NA) that were targeted for intracellular recordings. Abbreviations: rVRG and cVRG, rostral and caudal ventral respiratory groups which also contain neurons of the respiratory CPG; 5 and 7, trigeminal and facial motor nuclei; 7n, facial nerve; SO, superior olivary nuclei. **B**. Example of histological reconstruction (confocal fluorescence images) of recorded inspiratory neuron (ramp-I) in the preBötC region ventral to motoneurons of NAsc (semi-compact division) labeled by immunostaining (red) for choline acetyltransferase (ChAT). The locations of rhythmic neurons used for our analyses were routinely verified by filling the cells with neurobiotin by iontophoresis at the end of recording and fluorescently stained (see Methods), and in some cases further processed for immuno-identification of neurotransmitter phenotype (see ***Supplemental Figure 2***). The immunostaining for ChAT was used as a landmark for regional recording locations, and also to verify that recordings were obtained from interneurons and not motoneurons. A higher magnification image of neuronal morphology with background removed is shown in the dashed box at right. **C**. Example of histological reconstruction (confocal images) of a recorded expiratory neuron (aug-E) and fluorescently stained in the BötC region ventral to motoneurons of NAc (compact division) labeled by immunostaining (red) for ChAT. Higher magnification image of labeled neuron (background removed) is shown at right.

bioRxiv preprint doi: https://doi.org/10.1101/2024.08.12.607656; this version posted August 13, 2024. The copyright holder for this preprint (which was not certified by peer review) is the author/funder. This article is a US Government work. It is not subject to copyright under 17 USC 105 and is also made available for use under a CC0 license.

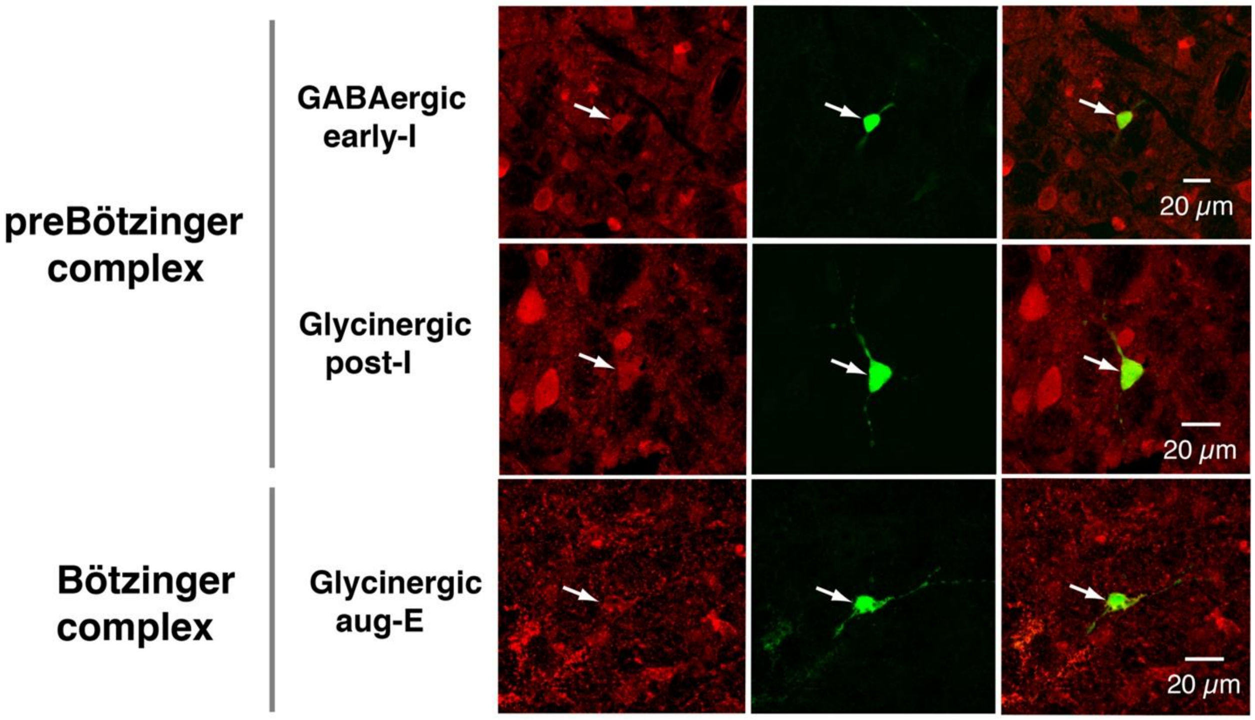

Supplemental Figure 2. Identification of glycinergic or GABAergic inhibitory neurons. Examples from separate experiments of neurons filled with neurobiotin during the somatic intracellular recordings, and subsequent immunohistochemistry with antibodies for glycine or GABA (red fluorescence), and Alexa Fluor 488-Avidin D conjugate to label neurobiotin. Images shown for the labeled respiratory neuron types in the ventrolateral medullary regions indicated are fluorescence single-plane confocal images for the glycine or GABA antibody labeling (left panels), neurobiotin labeling (middle), and superposition images showing double labeling (right). The single imaging planes shown were chosen to optimize visualization of co-labeling of the somata of the recorded neuron in each case.

bioRxiv preprint doi: https://doi.org/10.1101/2024.08.12.607656; this version posted August 13, 2024. The copyright holder for this preprint (which was not certified by peer review) is the author/funder. This article is a US Government work. It is not subject to copyright under 17 USC 105 and is also made available for use under a CC0 license.

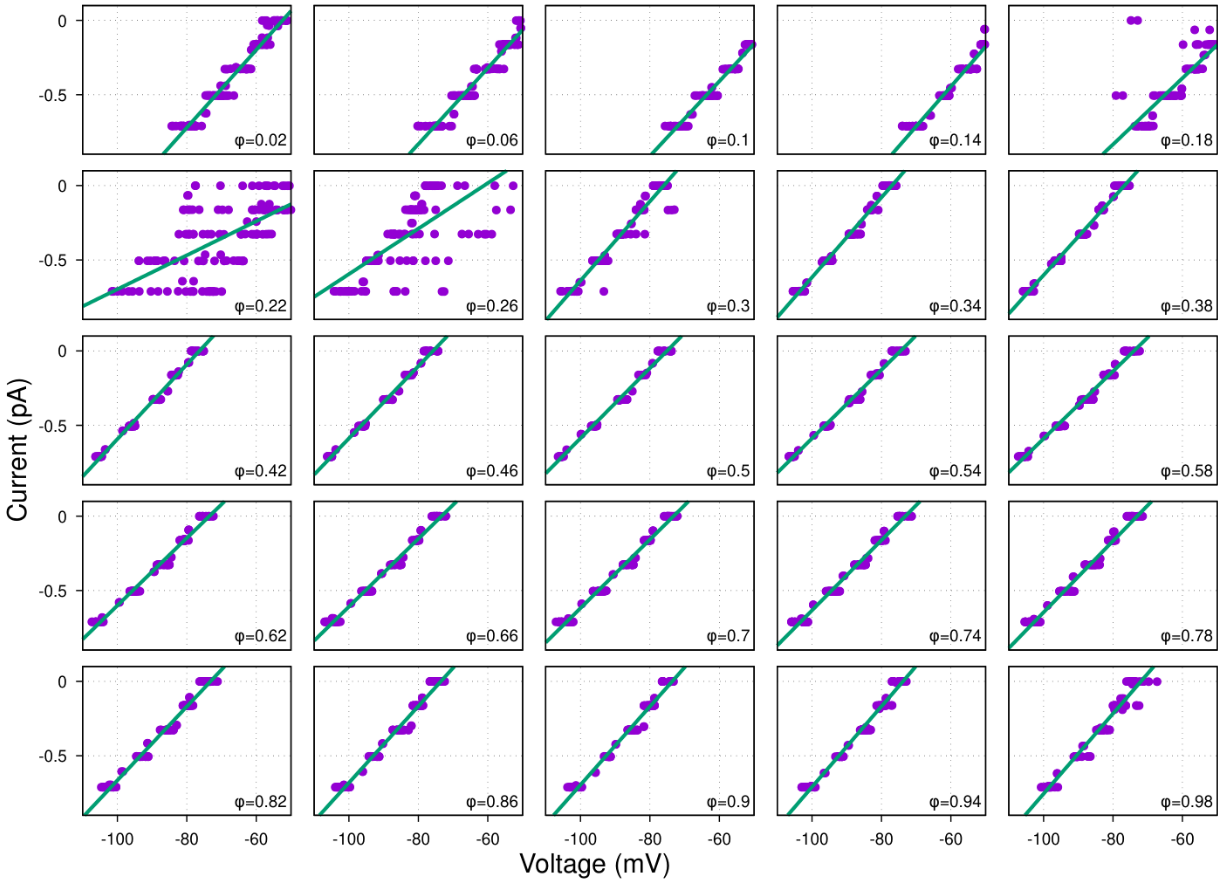

Supplemental Figure 3. Linear regressions of the injected current vs. membrane voltage for different phases of the respiratory cycle. Shown are the injected current (y-axis) vs. membrane voltage (x-axis) relations for 25 different phases of the respiratory cycle from a current-clamp recording from a ramp-I preBötC neuron. The phase value is indicated in the bottom right corner of each plot. This figure illustrates that the phase-specific current-voltage dependencies are almost perfectly linear in a wide range of voltage values. The exception is the interval of phase values near the transition from inspiration to expiration (0.2-0.25 for this particular recording) which reflects some cycle-cycle variability of the inspiratory phase durations. Less perfect linear fits near the transitions between respiratory phases manifest as bumps in the error estimates shown by black lines in ***Figures 1D, 3, 5, 6, 7***.
